# Supplementary material for: Multi-dimensional evaluation of user-operated audible contrast sensitivity tests towards efficient hearing healthcare services
Source: PLOS Digit Health. 2026 Jul 13;5(7):e0000975. doi: 10.1371/journal.pdig.0000975 (PMC13362146; doi:10.1371/journal.pdig.0000975)
Supplement: Attachment — Submitted filename: Response Letter_R1.pdf [file pdig.0000975.s002.pdf]

January 2026

Dear Dr. Michael Winter and Dr. Leo Anthony Celi

Thank you for processing our Manuscript PDIG-D-25-00510 entitled "Evaluating user-operated tests of audible contrast sensitivity towards efficient hearing healthcare services". Based on your feedback, we believe we have made the necessary improvements to make this manuscript acceptable and be published in PLOS Digital Health. This letter contains our answer to the editorial office and reviewers' comments point-by-point.

The original concerns/comments are in *italic blue.*

The replies are in black.

The changes in the manuscript are provided in *italic grey.*

Please note that the reference numbers in this letter correspond to the reference list provided at the end of this document, rather than the references in the manuscript.

We have kept the structure as in the original article (i.e., the methods section at the end) as it is the one indicated in the Authors' guidelines. However, we have included a new section "Experimental design *and study framework*" between the introduction and the results to improve the readability and flow of the manuscript.

Sincerely,

Raul Sanchez-Lopez on behalf of the authors.

To the Editorial office:

Reviewer #1: This research and invention focused on overlooked area of care, and it has the potential to significantly enhance the accessibility of hearing loss diagnosis and treatment. I, therefore, understand the significance of this paper. The paper is crafted carefully, and the experiments were conducted systematically and thoroughly. However, there are critical issues that are missed in this paper.

Thank you for taking the time to carefully evaluate our manuscript.

First, order or presentation make the paper difficult to understand and fully evaluate. the methodology should come before the results, discussion and conclusion, which makes reading and comprehension of the paper easier. However, on this paper the methodology was the last part of the paper.

The structure of the manuscript is in accordance with the authors' guidelines, i.e., with the methods section at the end. However, we have reviewed carefully the manuscript to make the flow easier to follow despite the methodology still being placed at the end. We have moved several parts of the text to a new section "Experimental design and study framework" which introduces the fundamental aspects of the methodology before the results section.

Changes:

Between the Introduction and the Results, we have added a new section named "Experimental design and study framework". In this new section, we introduce the protocol and we have moved the subsections about the participants, test benchmarks, and test candidates from the results to this section.

Second, the sample technique wasn't explicitly mentioned, and it is not clear what measures were taken to ensure the randomness of the sample.

In the study, participants were recruited via purposive convenience sampling from a database of previous research volunteers at the Technical University of Denmark. To ensure a diverse range of cognitive and auditory characteristics, we targeted specific counts for normal-hearing controls and hearing-impaired subgroups based on their RDS scores.

Change:
In the method section: "The potential participants of the study were recruited via purposive convenience sampling from the database of the Hearing Systems Section of

the Technical University of Denmark (DTU). In the recruitment process, care was taken to ensure [...]"

Third, there were arbitrary thresholds without sufficient justifications. For example, in 1-button task, 1.6 second was used to categorize miss and hit, however, there is no sufficient explanation why this number was selected and what was the investigator try to achieve by timing the tasks. In addition, subsequent tasks, such as yes/no task and 3-AFC tasks, weren't timed, but adequate justifications weren't provided why these tasks weren't timed.

The 1-button task employed a running-stimulus paradigm consisting of consecutive 1-second stimuli. These stimuli were either a reference sound ('wave') or a target sound ('ambulance'). Participants were instructed to respond only upon detecting a target; a lack of response was recorded as a non-detection. To facilitate automated scoring and allow an adaptive procedure, a fixed response window was implemented to categorize each trial as either a HIT or a MISS. Since this is the same paradigm as the manual ACT, we assumed that the response window would be comparable. The response times for the other two tasks were recorded but did not have an effect on the procedure.

Change

For the 1-button task the following text has been added: "We implemented a fixed response window (starting 200 ms after stimulus onset and ending 600 ms after offset, for a total of 1.6 s) to categorize trials as HIT or MISS. This window mirrors the parameters used in the manual ACT, assuming comparable response times between physical buttons and touch screens."

For the tasks Yes/No and 3AFC, this text has been added: "There were no restrictions on response time since the stimulus was presented sequentially".

Fourth, the discussion section of the paper was too sparse and the findings of the experiments weren't deeply explored or explained further in the context of existing literature. I also think the diversity of the participants, real clinical setting testing and the use of multi-dimensional protocols was the strength of the paper.

Thank you for pointing this out. We have revised the discussion and modified various paragraphs. One of the main changes is the discussion of the previous studies and subjective evaluations. Besides, we have added explanations connected to some of the limitations to provide more context.

Change in manuscript:

Discussion of previous studies:

For example, a spectral ripple discrimination test was developed and further optimized for clinical applications[1,2]. However, there is no clinical evidence of the use of the test for aural rehabilitation. Evaluations of the STM task in the Portable Automated Rapid Testing (P.A.R.T)[3] platform by Lelo de Larrea-Mancera, Gallun and colleagues initially focused on stimulus generation and population-specific performance[4,5]. Subsequent research has significantly expanded this scope to include formal psychoacoustic modeling via the Adaptive Scan algorithm to improve time efficiency and rigorous usability testing to ensure the platform's viability for at-home auditory assessment[5,6]. In contrast, we have directly tested U-ACT in a realistic environment, with a diverse sample in terms of auditory and cognitive abilities.

The evaluation methods of user-operated audiological tests are mainly focused on accuracy and reliability[7]. The automated pure-tone audiometry protocol utilized in the UAud project (AMTAS) is supported by a substantial body of evidence validating its reliability and clinical effectiveness [8]. Previous multi-center studies have demonstrated high correlation between AMTAS-derived thresholds and traditional manual audiometry[9], including validations of "at-home" settings[10]. Furthermore, the UAud project has examined the impact of AMTAS results on hearing-aid fitting outcomes [11]. However, comprehensive usability data remain scarce, particularly regarding user experience of older adults. This gap is critical, as cognitive decline may significantly and negatively impact the successful completion of user-operated diagnostics in the overwhelmingly elderly target population. The present study addresses this by including older adults with lower cognitive abilities to determine if U-ACT is accessible to a broad population regardless of cognitive profile, evaluating also the use of instructions and the quality of interaction for five alternative test variants. To our knowledge, this is the first study in audiology to select and evaluate an auditory test using a multimodal approach centered on the user experience and guided by the RAMES framework.

Discussion Subjective evaluation:

Subjective evaluations are known to have response biases and systematic errors that can distort survey outcomes. In our study, we chose a visual analogue scale because it offers higher sensitivity to small changes than discrete scales and provides continuous data suitable for more powerful statistical analysis[12]. However, it is known to have leniency bias, end-aversion bias, and directional bias (such as pseudoneglect)[13]. The challenge is to control how the participants make use of the scale to ensure they accurately translate their sensory experiences into the linear format without clustering responses or reinterpreting anchors.

In our study, the inclusion of both positive and negative item wordings was a deliberate methodological choice designed to mitigate acquiescence bias (the tendency of

respondents to agree with statements regardless of their content) and directional response bias. By employing dual formulations for the same attribute, we sought to enhance the internal validity of the subjective ratings. A significant difference between U-ACT candidates across both wordings thus indicates a robust perceived difference in usability. In contrast, when the wording factor itself is significant, it highlights a framing effect, suggesting that participant judgments are sensitive to how the question is posed. Furthermore, by doubling the observations per attribute through these dual formulations, we increased the statistical power of the usability evaluation.

The response bias has been investigated in several studies and different mitigation approaches have been tried, such as using intermediate ticks in the scale [14], combination with other techniques[13], or complex statistical modelling[15]. In our study, the combination of standardization of the data and the redundancy obtained through use of different wording allowed us to identify subtle differences between the test candidates.

Discussion Limitations:

While the multi-dimensional evaluation was valuable, there are limitations to consider. Firstly, participants were sourced from the university's database, potentially biasing results due to prior hearing research experience affecting task interactions. Besides, the cognitive screening was only based on one aspect of a specific ability (i.e., working memory). Although other types of assessments were considered[16,17], we decided to use a robust test that has been previously used at DTU in various studies[18], rather than a test including aspects like attention or executive function that could be time consuming or less reliable. Secondly, repeated assessments of the same listening ability within each block may have positively influenced results through training effects. This was a known limitation that we could not disentangle in the analysis. As an alternative, we could have used a different experimental design such as a randomized trial with one group per test candidate, however, this approach would have resulted in other limitations since each individual would only have used one of the candidates. Additionally, the protocol lacked speech intelligibility in noise tests as a reference measure, as included in Zaar et al.[19]. The U-ACT test is expected to be equivalent to the clinical ACT and have a similar predictive power so it can be considered a proxy for speech-in-noise tests. However, this should be explored in a separate study comparing the two contrast tests with the hearing-in-noise test with a clinically relevant population.

Reviewer #2: Thank you for giving me the opportunity to review your article.

1. The authors mentioned that there were "Some insights emerged, such as perceived differences in test difficulty and length among candidates, and a difference in results based on attribute wording which had a strong and clear significance in user evaluations." (line 331). It might be worth elaborating if these differences were the result of candidates having different hearing/cognitive abilities, or if the distribution of candidates who have different experiences in test difficulty and length is similar across the different hearing/cognitive abilities groups.

Thank you for pointing this out. In the statistical modelling part, we indeed used a different model consisting of: *Attribute = group + candidate + wording*

However, group was not significant and was removed from the model.

Change:

"Subsequently, linear models were created for each attribute in the following format: Attribute = group + candidate + wording. However, the group factor was not significant in any of the attribute models and was thus removed. "

2. The authors mentioned that the participants had an option to "Call the examiner: The user always had the option to call the examiner for task explanation". It might be worth stating whether any participants called the examiner for help, and if it the calls for help affected the rating of the test experience.

Thank you for your comment. Eleven participants used the "Call the examiner" button in *block 1*. Although it would be interesting to check whether these participants had a worse test experience, no evaluation was collected in *block 1*. As the three levels of support are indicative of the difficulty of understanding the test we have added details of these data in the results section.

Change:

The following text has been added to the results: "Beyond the primary success rates, participants frequently utilized support features during the first block. In 31% of the test runs (44/140), participants accessed written instructions; 18% (25/140) accessed additional visual cues, and 11% (15/140) requested assistance from the examiner. The majority of 'call the examiner' requests were attributed to user interface (UI) design at

the end of the demonstration phase, where some participants repeated the demo multiple times after failing to locate the 'start test' button. By the second visit (Block 3), support requirements decreased: written instructions were accessed in only 13% of runs (18/140) and visual cues in 5% (7/140), with zero instances of examiner intervention."

Reviewer #3: This paper develops and evaluates user-operated alternatives to the ACT test. While it is relevant to PLOS Digital Health and the research overall does seem sound, I have several significant issues with the work that I don't think can be addressed with a revision. Specifically, I think the paper needs significant rewrites, and additional experiments need to be run before I can recommend the paper for publication. Please note that the examples I use to illustrate my points are not exhaustive; there are usually more cases of the issues I point out.

Thank you for taking the time and effort to review our work and preparing this comprehensive report.

Poor writing and organization:
First, this paper is rather difficult to follow at both a structural and a sentence level. Regarding the latter, while sentences are not grammatically incorrect, there is a lot of awkward phrasing that makes for a choppy and, at times, verbose read. Just taking the sentence beginning at the end of line 409 as an example, "We aimed at a total of 30 participants where there would be a control group formed of 6 young NH listeners (<30 years old), a group of people with hearing loss and with high cognitive abilities (i.e., higher scores in the RDS test, HIhi.), and another group of participants with hearing loss with lower cognitive abilities (i.e., lower scores in the RDS test HIlo)" could much more cleanly rephrased as "We aimed to recruit 30 participants for this study. This comprised 6 NH listeners, 12 listeners with hearing loss and high cognitive abilities (Hihi), and 12 listeners with hearing loss and low cognitive abilities (Hilo). Cognitive ability was determined based on RDS scores, with the high group scoring between 14 and 21 and the low group scoring between 6 and 12."

Thank you for your comment and this specific example. We have revised this sentence accordingly and have also revised the rest of the manuscript to improve the readability.

Change (example):
"The study aimed to include a total of 30 participants, stratified into three groups: six young NH controls, twelve HI listeners with high cognitive abilities (HI_hi), and twelve HI listeners with low cognitive abilities (HI_lo). Cognitive classification was based on

Reverse Digit Span (RDS) scores; the high-ability group included scores from 14 to 21, while the low-ability group included scores from 6 to 12."

Secondly, definitions for concepts that need to be explained frequently come after their use, so I had to read the sentence, find the definition, then go back and read the sentence again to understand what the authors were getting at. One example is lines 46-52, where the authors introduce the ACT test as a version of the STM test, neither of which is really introduced or defined (more on this later). They both involve speech reception thresholds, which are defined in the next sentence, meaning that a reader has to start this whole section over again to understand what they are reading, with the context from the later sentences.

We have included a paragraph explaining previous research with STM detection tests so the introduction of ACT is not disrupted and the reader has access to the necessary background information and understand the rationale of using this test.

Change:
In the introduction:

One promising approach involves the detection of spectro-temporal modulations (STM) imposed on broadband sounds[20–22]. These modulations create spectral and temporal ripples that mimic the rapid fluctuations of natural speech. A key advantage of STM stimuli is the precise control of modulation levels within classical psychoacoustic adaptive procedures[22,23]. This paradigm is analogous to contrast sensitivity testing in the visual domain, which utilizes spatial and temporal patterns[24,25]. Because the ability to discriminate subtle cues in noisy speech is strongly associated with STM sensitivity[26,27], these tests have been proposed both as a proxy for speech-in-noise perception in ecologically valid environments and as a predictor of hearing-aid benefit[19].

As another example, on line 91, RDS scores are introduced, but it's only in the Methods that they are noted as a test for cognitive ability (lines 407-408). As a final example here, the RAMES framework is mentioned for the first time on line 356, and even the acronym is not spelled out until line 391, where it's just cited and not defined. I bring up these sentence-level issues to highlight the structural issues with the paper, namely that it's not particularly accessible to readers who aren't intimately familiar with this specific area of research.

Thank you for pointing this out – point well taken. We have introduced both concepts earlier in the text and made an effort to rectify similar problems at other places.

Change:

The Reversed Digit Span test is now introduced in the Experimental design section: "To assess the audiological characteristics, the participants underwent standard pure-tone audiometry as part of the protocol. Furthermore, their cognitive abilities were assessed using the reversed digit span[18,28] (RDS) test, which specifically evaluates the working memory performance of the participant. "

The RAMES framework is now introduced in the Experimental design section.

In general, it seems as though the authors struggled to write a paper where the results preceded the methods, as I frequently had to go to the Methods section to understand what was being presented in the results (this is highlighted by the fact that several times the authors stated "(see Methods section)" after portions of the Results section) and the Results section is frequently bloated by half descriptions of the Methods employed to achieve the Results. As examples, lines 87-90, 91-93, 116-122, and the entire section starting from 146 are Methods, not results. The authors need to go back and firmly delineate what goes into Methods and what goes into Results.

Thank you for your suggestions. We have moved part of the results to a new section entitled "Experimental design and study framework" which introduces the fundamental aspects of the methodology before the results section.

Change:
New section "Experimental design and study framework"

In general, I feel like the paper needs a significant rewrite for clarity. In particular, I would recommend that the authors read other papers that place the Results section before the Methods section and pay particular attention to how information is introduced and which sections contain which information. One particular point of emphasis: the authors really should clearly introduce the ACT and STM tests in the introduction, as these are foundational to their work, and without a clear understanding of those, readers who are not familiar with them are going to have a frustrating experience.

This comment relates to another earlier comment. As stated in response to that earlier comment, we have included a paragraph explaining previous research with STM detection tests so the introduction of ACT is not disrupted and the reader can have a background and understand the rationale of using this test.

Study population issues:

I also have issues with the study population. I can understand why the population of hearing-impaired participants is low, as I can see how that would be a more difficult population to recruit. However, there are only 6 individuals who comprise the control group, and the only requirement for being a part of the control group is that they are below 30 years old and do not have hearing loss. There's no reason for this group of participants to be that low, especially given that this study was conducted in a university setting. The authors should conduct the study with additional control group participants and re-run their analysis. Furthermore, is age just a proxy for hearing ability? Was one of the requirements for being in the hearing impaired group being over 50, and one of the requirements for being in the normal hearing group being under 30? Or was this just how recruitment panned out? The authors should clarify whether they recruited specifically for age or not, and if they did, they should justify why they did so.

In the study, we aimed at a total sample of 30 individuals. However, two participants dropped out after the first visit. The NH group is intentionally small because we only expect small differences among them that are not clinically relevant. According to Nielsen & Landauer (1993), the user-centric evaluations often make use of 5-20 participants depending on the complexity of the test. Therefore, our sample size of 6 Normal Hearing (NH) listeners is sufficient to establish a stable baseline for interaction quality, as the mathematical model for interface debugging shows that the vast majority of usability and interaction problems are identified within the first 3 to 5 participants.

The age is not a proxy for hearing or cognitive abilities; both aspects were tested also in the NH group. However, in this study, we aimed to have a sample of 22-24 hearing-impaired listeners with cognitive and auditory variability with a balance in terms of gender and age.

Change

"We intentionally recruited 6 NH listeners below 30 years old which is sufficient to establish a stable baseline for interaction quality [29]."

Also, I have several issues with the inclusion of cognitive abilities. Why was this a point of emphasis? The authors should justify this with citations in the introduction. Also, why are RDS scores a fair evaluation of cognitive abilities? To me, this is a rather narrow view of cognitive ability, and there are many other tests; the authors need to justify the use of this.

We agree with the reviewer that working memory is only one aspect of cognition. However, the positive manifold is a central, highly replicated finding in psychology where all cognitive ability tests, exhibit positive correlations with one another. We

utilized RDS because it was the standard test used at DTU for screening patients. However, we discuss the inclusion of other tests or screening assessments like Mini mental or MoCA. Diversity of cognitive abilities of the test participants was indeed a focus point, as we expected cognitive abilities to be associated with success/failure with the test instructions.

Regarding the citations in the introduction, we have added:

Mata, R., Schooler, L. J. & Rieskamp, J. The aging decision maker: Cognitive aging and the adaptive selection of decision strategies. *Psychol. Aging* **22**, 796–810 (2007).

Contreras-Somoza, L. M. *et al.* Usability and User Experience of Cognitive Intervention Technologies for Elderly People With MCI or Dementia: A Systematic Review. *Front. Psychol.* **12**, 636116 (2021).

Change:

We have added to the limitations:

Firstly, participants were sourced from the university's database, potentially biasing results due to prior hearing research experience affecting task interactions. Besides, the cognitive screening was only based on one aspect of specific ability (i.e., working memory). Although other types of assessments were considered[60,61], we decided to use a robust test that has been previously used at DTU in various studies[38], rather than a test including aspects like attention or executive function that could be time consuming or less reliable.

A note on the language here. The statement "Since one of the study's goals was to obtain an evaluation from a diverse group of participants with varying hearing and cognitive abilities..." is confusing to me. Which of the five goals the authors laid out does this refer to? My best guess is Goal 4, but if that's the case, evaluation on a diverse group of participants is a means of ensuring Goal 4; the goal itself is not to evaluate on a diverse group of participants. Really, I think that whole sentence can be removed, and the section can simply begin with something like "Figure 1 shows the results from..." which removes some of the Methods language from the Results section as I noted earlier.

The statement has been removed and the paragraph moved to the Experimental design ... section

Change:
The sentence has been removed.

Also, the statement "We aimed at a total of 30 participants where there would be a control group formed by 6 young NH listeners" (lines 409-411) is confusing. Why did the authors aim to recruit only 30 participants? Why specifically 6 participants? Is this just a language issue, where a statement such as "We recruited a total of 30 participants that comprised 6 young NH listeners..." is what the authors meant?

This has been resolved above.

Introduction issues:
The introduction generally lacks citations. There are only 10 citations throughout, and several claims really should be backed up with citations. For instance, this sentence: "The currently used audiometric tests, which are time-consuming and require active participation of a hearing care professional, represent a bottleneck that effectively limits access to hearing healthcare." (lines 26-28), have health care professionals or patients noted that they are time consuming and represent a bottleneck?

Change:

This sentence is supported by the following citations now:
4. Garuccio, J., Ukert, B., Arnold, M., Phillips, S. & Pesko, M. F. Using Supply and Demand to Identify Shortages in the Hearing Health Care Professional Workforce. JAMA Otolaryngol. Neck Surg. 151, 868 (2025).

5. Dillard, L. K. et al. Service delivery approaches related to hearing aids in low- and middle-income countries or resource-limited settings: A systematic scoping review. PLOS Glob. Public Health 4, e0002823 (2024).

6. Margolis, R. H. & Morgan, D. E. Automated Pure-Tone Audiometry: An Analysis of Capacity, Need, and Benefit. Am. J. Audiol. 17, 109–113 (2008).

Also, this sentence: "However, there has been a growing focus on additionally evaluating supra-threshold abilities, such as the patient's ability to discriminate speech in the presence of noise when the speech is fully audible." (lines 38-40), are there any statistics/studies to show this shift in focus?

We do not know of any statistics related to this, but there is a commonly mentioned need in the audiological community for such measures that has been the subject of numerous research efforts over the past decades. We have selected the publication by Plomp in 1978: "Auditory handicap of hearing impairment and the limited benefit of hearing aids", which inspired a shift in focus as suggested in Houtgast and Festen

(2008): “On the auditory and cognitive functions that may explain an individual’s elevation of the speech reception threshold in noise”, published 30 years later.

Change:

We have included the references to Plomp (1978) and Houtgast and Festen (2008) in the introduction.

Also, this sentence: “This has motivated the search for alternative tests capable of capturing the fundamental abilities employed in challenging 'cocktail-party' scenarios, which are characterized by multiple interfering sound sources including speech.” (lines 43-45), are there other studies that have developed and evaluated alternative tests?

We have included more references to studies on this topic.

Change:
The Influence of Cochlear Mechanical Dysfunction, Temporal Processing Deficits, and Age on the Intelligibility of Audible Speech in Noise for Hearing-Impaired Listeners - Peter T. Johannesen, Patricia Pérez-González, Sridhar Kalluri, José L. Blanco, Enrique A. Lopez-Poveda, 2016. https://journals.sagepub.com/doi/full/10.1177/2331216516641055.

Strelcyk, O. & Dau, T. Relations between frequency selectivity, temporal fine-structure processing, and speech reception in impaired hearing). J. Acoust. Soc. Am. 125, 3328–3345 (2009).

Also, I think it might make the introduction stronger if the first sentence was separated into multiple sentences; the authors could include actual statistics on the scope and impact of hearing loss to really emphasize the need for improving accessibility and reducing cost.

Thank you for pointing this out. We have rewritten the beginning of the introduction to be more precise about the context.

Change

The total number of people living with some degree of hearing loss worldwide is projected to rise from approximately 1.57 billion in 2019 to 2.45 billion by 2050. This represents an increase of 56.1% more individuals who will need hearing healthcare services[30]. Furthermore, it is expected that more than 44% of individuals aged 70 years and older will experience hearing deficits. With this perspective, and an increasing workforce shortage in hearing healthcare[31], there exists an urgent need to improve efficiency and reduce costs in hearing healthcare to ensure access to quality services [30,32,33]. The currently used hearing tests require active participation of a hearing care

professional, and represent a bottleneck that effectively limits access to hearing healthcare[34]. To meet the demand for hearing assessments, the availability of well-trained audiologists and clinical facilities is crucial[35]. However, audiologists are responsible for various important tasks beyond hearing diagnostic tests such as hearing-aid fitting and verification as well as counselling[36].

Figure issues:
I don't think the combination of the text, Figure 3, and Figure 3's caption does a good job of explaining the differences between the test candidates. The authors should rework these to make it clearer what is changing between each candidate. In particular, I was looking to see if only one thing had changed between candidates, to understand what variable was being tested with each candidate, and it seems like that's the case? I found this hard to follow.

Figure 3 represents a visual comparison of the U-ACT candidates that we believe is more effective than a table for most of the readers. The differences between the 5 test candidates are not only one aspect. Thus, it is more challenging to efficiently explain the differences.

Change:
We have simplified the caption and text explaining the figure:
"Figure 1 depicts the five U-ACT candidates showing their progression from quick-and-simple ACT-like (U-ACT-0, blue background), to the research-oriented STM-like (U-ACT-4, orange background) designs. The three intermediate candidates (green background) differ only in their task configuration. Note that the 1-button task is only compatible with a continuous stimulus."

For Figures 5-6, there's quite a bit going on, and a lot of significance between various groups. Fundamentally, I don't understand why positive and negative wordings were used; I appreciate that phrasing of statements can affect ratings, but there's no meaningful follow-up on how this should influence how I interpret participants' views on the candidates. For example, if I'm understanding correctly, in that first panel in Figure 5, there is a significant difference in the difficulty of candidates 2 and 3 when negative wording is used, but the difference is not significant when positive wording is used. So, is candidate 2 better in terms of difficulty? It just seems like these figures are introducing a lot of complexity, and all that the authors are getting out of it is a muddier picture. I will note that the point of a Discussion section is to contextualize results, and I was looking for some clarity there, but I did not find it. Also, I don't understand what the word

“wording” in the bottom left of each panel means. It also seems to cover up some data points in some panels.

The inclusion of both positive and negative item wordings was a deliberate methodological choice designed to mitigate acquiescence bias—the tendency of respondents to agree with statements regardless of their content—and directional response bias. By employing dual formulations for the same attribute, we sought to enhance the internal validity of the subjective ratings. This difference in the items is what we refer to as “wording”. A significant difference between U-ACT candidates across both wordings indicates a robust perceived difference in usability. In contrast, when the 'wording' factor itself is significant suggests that participant judgment is sensitive to how the question is posed. We have clarified this interpretation in the revised Discussion to show how these results identify the most 'user-robust' candidates.

Change:

The inclusion of both positive and negative item wordings was a deliberate methodological choice designed to mitigate acquiescence bias (the tendency of respondents to agree with statements regardless of their content) and directional response bias. This difference in the items is referred to as “wording”. By employing dual formulations for the same attribute, we sought to enhance the internal validity of the subjective ratings. A significant difference between U-ACT candidates across both wordings indicates a robust perceived difference in usability. In contrast, when the 'wording' factor itself is significant, it highlights a framing effect, suggesting that participant judgment is sensitive to how the question is posed.

Other issues:
Line 84: “A diverse group of participants” is a weird title for a subsection. I would recommend rephrasing to something like “Study participants”
Changed as suggested.
Lines 87-93, the language is a bit confusing here. Initially, I read it as 28 HI participants were recruited, and an additional 6 NH participants were recruited, which made the division into 11 and 11 confusing. Please clean up the language here.
Changed as suggested.

Figure 1 would be cleared if Right and Left were Right Ear and Left Ear.

Changed as suggested.

Line 105, "two groups" should be "two HI groups"
Changed as suggested.

Line 193, how many users were used for this refinement? These users were separate from the 28 participants that were used to evaluate the system, right?
Changed as suggested.

Line 435, what iPad model was used?
8th generation, MYL92KN/A

References used in the Response letter

1. Aronoff JM, Landsberger DM. The development of a modified spectral ripple test. *J Acoust Soc Am*. 2013;134(2):EL217-EL222. doi:10.1121/1.4813802

2. Landsberger DM, Dwyer RT, Stupak N, Gifford RH. Validating a Quick Spectral Modulation Detection Task. *Ear Hear*. 2019;40(6):1478. doi:10.1097/AUD.0000000000000713

3. Lelo de Larrea-Mancera ES, Koerner TK, Bologna WJ, et al. At-Home Auditory Assessment Using Portable Automated Rapid Testing (PART) to Understand Self-Reported Hearing Difficulties. *Trends Hear*. 2025;29:23312165251397373. doi:10.1177/23312165251397373

4. Lelo de Larrea-Mancera ES, Stavropoulos T, Hoover EC, Eddins DA, Gallun FJ, Seitz AR. Portable Automated Rapid Testing (PART) for auditory assessment: Validation in a young adult normal-hearing population. *J Acoust Soc Am*. 2020;148(4):1831-1851. doi:10.1121/10.0002108

5. Lelo de Larrea-Mancera ES, Stavropoulos T, Carrillo AA, et al. Remote auditory assessment using Portable Automated Rapid Testing (PART) and participant-owned devicesa). *J Acoust Soc Am*. 2022;152(2):807-819. doi:10.1121/10.0013221

6. Lelo de Larrea-Mancera ES, Stavropoulos T, Carrillo AA, et al. Validation of the adaptive scan method in the quest for time-efficient methods of testing auditory processes. *Atten Percept Psychophys*. 2023;85(8):2797-2810. doi:10.3758/s13414-023-02743-z

7. Shojaeemend H, Ayatollahi H. Automated Audiometry: A Review of the Implementation and Evaluation Methods. *Healthc Inform Res*. 2018;24(4):263. doi:10.4258/hir.2018.24.4.263

8. Margolis RH, Glasberg BR, Creeke S, Moore BCJ. AMTAS® : Automated method for testing auditory sensitivity: Validation studies. *Int J Audiol*. 2010;49(3):185-194. doi:10.3109/14992020903092608

9. Eikelboom RH, Swanepoel DW, Motakef S, Upson GS. Clinical validation of the AMTAS automated audiometer. *Int J Audiol*. 2013;52(5):342-349. doi:10.3109/14992027.2013.769065

10. Margolis RH, Killion MC, Bratt GW, Saly GL. Validation of the Home Hearing Test™. *J Am Acad Audiol*. 2016;27(5):416-420. doi:10.3766/jaaa.15102

11. Pedersen C, Pedersen ER, Laugesen S, et al. Comparison of hearing-aid effectiveness based on user-operated versus traditional audiometry: a randomised clinical trial. *Int J Audiol*. Published online December 2, 2024:1-8. doi:10.1080/14992027.2024.2434897

12. Voutilainen A, Pitkäaho T, Kvist T, Vehviläinen-Julkunen K. How to ask about patient satisfaction? The visual analogue scale is less vulnerable to confounding factors and ceiling effect than a symmetric Likert scale. *J Adv Nurs*. 2016;72(4):946-957. doi:10.1111/jan.12875

13. Sung YT, Wu JS. The Visual Analogue Scale for Rating, Ranking and Paired-Comparison (VAS-RRP): A new technique for psychological measurement. *Behav Res Methods*. 2018;50(4):1694-1715. doi:10.3758/s13428-018-1041-8

14. García-Pérez MA, Alcalá-Quintana R. Accuracy and precision of responses to visual analog scales: Inter- and intra-individual variability. *Behav Res Methods*. 2023;55(8):4369-4381. doi:10.3758/s13428-022-02021-0

15. Kersten P, White PJ, Tennant A. Is the Pain Visual Analogue Scale Linear and Responsive to Change? An Exploration Using Rasch Analysis. *PLOS ONE*. 2014;9(6):e99485. doi:10.1371/journal.pone.0099485

16. Nasreddine ZS, Phillips NA, Bédirian V, et al. The Montreal Cognitive Assessment, MoCA: a brief screening tool for mild cognitive impairment. *J Am Geriatr Soc*. 2005;53(4):695-699. doi:10.1111/j.1532-5415.2005.53221.x

17. Diniz BSO, Yassuda MS, Nunes PV, Radanovic M, Forlenza OV. Mini-mental State Examination performance in mild cognitive impairment subtypes. *Int Psychogeriatr*. 2007;19(4):647-656. doi:10.1017/S104161020700542X

18. Fuglsang SA, Märcher-Rørsted J, Dau T, Hjortkjær J. Effects of Sensorineural Hearing Loss on Cortical Synchronization to Competing Speech during Selective Attention. *J Neurosci*. 2020;40(12):2562-2572. doi:10.1523/JNEUROSCI.1936-19.2020

19. Zaar J, Simonsen LB, Laugesen S. A spectro-temporal modulation test for predicting speech reception in hearing-impaired listeners with hearing aids. *Hear Res*. 2024;443:108949. doi:10.1016/j.heares.2024.108949

20. Bernstein JGW, Mehraei G, Shamma S, Gallun FJ, Theodoroff SM, Leek MR. Spectrotemporal Modulation Sensitivity as a Predictor of Speech Intelligibility for Hearing-Impaired Listeners. *J Am Acad Audiol*. 2013;24(4):293-306. doi:10.3766/jaaa.24.4.5

21. Chi T, Gao Y, Guyton MC, Ru P, Shamma S. Spectro-temporal modulation transfer functions and speech intelligibility. *J Acoust Soc Am*. 1999;106(5):2719-2732. doi:10.1121/1.428100

22. Zaar J, Simonsen LB, Dau T, Laugesen S. Toward a clinically viable spectro-temporal modulation test for predicting supra-threshold speech reception in hearing-impaired listeners. *Hear Res*. 2023;427:108650. doi:https://doi.org/10.1016/j.heares.2022.108650

23. Levitt H. Transformed up-down methods in psychoacoustics. *J Acoust Soc Am*. 1971;49(2):Suppl 2:467+.

24. Pelli DG, Bex P. Measuring contrast sensitivity. *Vision Res*. 2013;90:10-14. doi:10.1016/j.visres.2013.04.015

25. Dorr M, Wille M, Viulet T, et al. Next-generation vision testing: the quick CSF. *Curr Dir Biomed Eng*. 2015;1(1):131-134. doi:10.1515/cdbme-2015-0034

26. Mehraei G, Gallun FJ, Leek MR, Bernstein JGW. Spectrotemporal modulation sensitivity for hearing-impaired listeners: Dependence on carrier center frequency and the relationship to speech intelligibility. *J Acoust Soc Am*. 2014;136(1):301-316. doi:10.1121/1.4881918

27. Bernstein JGW, Danielsson H, Hällgren M, Stenfelt S, Rönnberg J, Lunner T. Spectrotemporal Modulation Sensitivity as a Predictor of Speech-Reception Performance in Noise With Hearing Aids. *Trends Hear*. 2016;20(0). doi:10.1177/2331216516670387

28. Blackburn HL, Benton AL. Revised administration and scoring of the Digit Span Test. *J Consult Psychol*. 1957;21(2):139-143. doi:10.1037/h0047235

29. Nielsen J, Landauer TK. A mathematical model of the finding of usability problems. In: *Proceedings of the SIGCHI Conference on Human Factors in Computing Systems - CHI '93*. ACM Press; 1993:206-213. doi:10.1145/169059.169166

30. Haile LM, Kamenov K, Svitil Briant P, et al. Hearing loss prevalence and years lived with disability, 1990–2019: findings from the Global Burden of Disease Study 2019. *The Lancet*. 2021;397(10278):996-1009,. doi:10.1016/S0140-6736(21)00516-X.

31. Shen Y, Zhou T, Zou W, et al. Global, regional, and national burden of hearing loss from 1990 to 2021: findings from the 2021 global burden of disease study. *Ann Med*. 2025;57(1):2527367. doi:10.1080/07853890.2025.2527367

32. Clark JL, Swanepoel DW. The World Report on Hearing – a new era for global hearing care. *Int J Audiol*. 2021;60(3):161-161. doi:10.1080/14992027.2021.1881318

33. Garuccio J, Ukert B, Arnold M, Phillips S, Pesko MF. Using Supply and Demand to Identify Shortages in the Hearing Health Care Professional Workforce. *JAMA Otolaryngol Neck Surg*. 2025;151(9):868. doi:10.1001/jamaoto.2025.2112

34. Dillard LK, Der CM, Laplante-Lévesque A, et al. Service delivery approaches related to hearing aids in low- and middle-income countries or resource-limited settings: A systematic scoping review. Robinson J, ed. *PLOS Glob Public Health*. 2024;4(1):e0002823. doi:10.1371/journal.pgph.0002823

35. Windmill IM, Freeman BA. Demand for Audiology Services: 30-Yr Projections and Impact on Academic Programs. *J Am Acad Audiol*. 2013;24(5):407-416. doi:10.3766/jaaa.24.5.7

36. Parmar BJ, Rajasingam SL, Bizley JK, Vickers DA. Factors Affecting the Use of Speech Testing in Adult Audiology. *Am J Audiol*. 2022;31(3):528-540. doi:10.1044/2022_AJA-21-00233
