# Supplementary material for: Multi-dimensional evaluation of user-operated audible contrast sensitivity tests towards efficient hearing healthcare services
Source: PLOS Digit Health. 2026 Jul 13;5(7):e0000975. doi: 10.1371/journal.pdig.0000975 (PMC13362146; doi:10.1371/journal.pdig.0000975)
Supplement: Attachment — Submitted filename: Response Letter_R2.pdf [file pdig.0000975.s003.pdf]

April 2026

Dear Dr. Michael Winter and Dr. Leo Anthony Celi

Thank you for processing our Manuscript PDIG-D-25-00510 originally entitled "Evaluating user-operated tests of audible contrast sensitivity towards efficient hearing healthcare services". Based on your feedback, we believe we have made the necessary improvements to make this manuscript acceptable for publication in PLOS Digital Health. This letter contains our point-by-point responses to the editorial office and the reviewers.

The original concerns/comments are in *italic blue.*

The replies are in black

The changes in the manuscript are in *italic grey*

Although, we tried to move parts of the method sections to an appendix, we have kept the structure as in the original resubmission R1. The modest reduction in length of the main manuscript did not outweigh the additional inconvenience for readers who would want a complete and coherent presentation of the methodology. Besides, we have added conclusions which are directly connected to the goals of the study as suggested by reviewer 3.

Please consider a small change in the title to: **Multi-dimensional evaluation of user-operated audible contrast sensitivity tests towards efficient hearing healthcare services** to stress the importance of the different types of evaluations (usability, reliability, psychoacoustics) included in the study.

Sincerely,

Raul Sanchez-Lopez on behalf of the authors.

Reviewer #1: The structure of the paper is improved significantly, enhancing readability of the study. Sentence level issues are also resolved, and enabled easy understanding of the paper. Compelling clarifications were provided with ample citations which strengthened the discussions on the paper. The technical parts are also adequately explained.

Thank you for your comments and your feedback on the revision.

Reviewer #2: I would like to thank the authors for their thoughtful and constructive response to the previous round of peer review.

Thank you for your comments and your feedback on the revision.

An aim of the study is to ensure that "the test should be accessible to a broad group of patients without requiring any special modifications for subpopulations, including those with low cognitive abilities.", Upon reviewing the section on participants recruited, there are participants who are hearing-impaired with both higher and lower cognitive abilities. However there seems to be only participants with normal hearing and higher cognitive abilities, and there seems to be a lack of participants with normal hearing abilities and lower cognitive abilities. It would be good if the authors could elaborate on the choice of participants, especially as a key strength of the study mentioned was inclusion of "a diverse participant selection regarding hearing losses and cognitive abilities".

Thank you!

Thank you for pointing this out. There were indeed no participants with normal hearing and lower cognitive abilities included. The reason is that the primary age group that would use the U-ACT test consists of adults above 60 years of age. This target group might experience hearing loss, contrast loss and cognitive decline. In contrast, the young normal-hearing group was added as a reference in the present study, not as a potential clinical population. However, it is of interest to explore the use of U-ACT in other populations in future studies. We have added this to the discussion.

L486-488: Furthermore, young normal-hearing listeners with low cognitive abilities might be an interesting population to evaluate the test in terms of reliability and usability.

Reviewer #3: The authors have done an excellent job of responding to reviewer feedback and making appropriate revisions. I'm generally satisfied with the updated manuscript, but I do have a couple of comments.

Thank you for your comments and your feedback on the revision.

1. While I appreciate the thought behind adding a new section after the introduction and before the results, it is effectively a methods section, and it is a bit strange to have two methods sections. I will note that while PLOS's submission guidelines do state that the preference is for the Methods section to come after the Discussion, it does state "To provide flexibility, however, authors are also able to include the Materials and Methods section before the Results section or before the Discussion section." Given there's quite a bit of context required to follow the Results section, I might recommend just combining the "Experimental design and study framework" section and "Methods" section into one section that follows the Introduction and precedes the Results. The Methods section is also quite long, and I would recommend considering moving non-essential definitions to supplemental materials. If the authors really would like to keep the methods after the discussion, I don't think adding a pseudo-methods section is the way to go; rather, the authors should work in the needed context into the introduction/results section.

We carefully considered moving parts of the Methods section to the Supplementary Material but ultimately decided to retain the full Methods in the main paper. The modest reduction in length of the main manuscript did not outweigh the additional inconvenience for readers who would want a complete and coherent presentation of the methodology.

2. A suggestion to improve readability: At the end of the introduction, the authors lay out five objectives, but these aren't referred to again after that. Given the number of results, it may help to refer back to these objectives in the results (and maybe also the discussion) so readers can quickly understand what objectives are being addressed by each finding. With this approach, I'd also recommend making the list of objectives in the introduction into an actual numbered list to give it the visual standout so that readers know to pay attention to it.

Thank you for this suggestion. We have modified the formatting of the list of objectives at the end of the introduction accordingly and refined the subheadings of the results that are related to the mentioned goals. In addition, we have added a concise and clear conclusion section to connect the findings with the goals.

L515-537: Conclusion

The present study utilized a multi-dimensional evaluation framework to compare user-operated audible contrast sensitivity tests in terms of feasibility, reliability, and accessibility towards the implementation of the U-ACT in hearing healthcare services.

- *The test proved easy to perform, patients immediately understood the instructions; this is supported by the fact that examiner interventions dropped to zero instances by the second visit and participants reported the highest "understanding" ratings for the preferred test paradigm, U-ACT-2.*
- *The test duration was not unnecessarily long; U-ACT-0 and U-ACT-2 were significantly more efficient than traditional methods, completing in an average of 90 and 202 seconds respectively, compared to over 324 seconds for the STM benchmark.*
- *The test results were reliable and repeatable, demonstrating excellent agreement with clinical benchmarks. This was specifically true for the U-ACT-2 Yes/No candidate which achieved an ICC of 0.84 (test-retest) and a high correlation with the manual ACT benchmark (ICC > 0.90).*
- *The test was accessible to a broad group of patients, with successful completion independent of participant subpopulation or cognitive profile. Statistical analysis verified that the cognition grouping did not significantly influence measured thresholds or the reliability of the results.*
- *The quality of interaction was optimal, enabling patients to perform the test seamlessly; subjective data confirmed the U-ACT-2 Yes/No candidate was perceived as the least difficult and most usable interface among all tested variants.*
