# Supplementary material for: Multi-dimensional evaluation of user-operated audible contrast sensitivity tests towards efficient hearing healthcare services
Source: PLOS Digit Health. 2026 Jul 13;5(7):e0000975. doi: 10.1371/journal.pdig.0000975 (PMC13362146; doi:10.1371/journal.pdig.0000975)
Supplement: Peer review history

# Peer review: Decision Letter 0

**DOI:** 10.1371/journal.pdig.0000975.r001

**Authors:** Zhao Ni, Michael Winter

1 Dec 2025

PDIG-D-25-00510

Evaluating user-operated tests of audible contrast sensitivity towards efficient hearing healthcare services

PLOS Digital Health

Dear Dr. Sanchez-Lopez,

Thank you for submitting your manuscript to PLOS Digital Health. After careful consideration, we feel that it has merit but does not fully meet PLOS Digital Health's publication criteria as it currently stands. Therefore, we invite you to submit a revised version of the manuscript that addresses the points raised during the review process.

Please submit your revised manuscript by Jan 30 2026 11:59PM. If you will need more time than this to complete your revisions, please reply to this message or contact the journal office at digitalhealth@plos.org. When you're ready to submit your revision, log on to https://www.editorialmanager.com/pdig/ and select the 'Submissions Needing Revision' folder to locate your manuscript file.

Please include the following items when submitting your revised manuscript:

* A rebuttal letter that responds to each point raised by the editor and reviewer(s). You should upload this letter as a separate file labeled 'Response to Reviewers'. This file does not need to include responses to any formatting updates and technical items listed in the 'Journal Requirements' section below.

* A marked-up copy of your manuscript that highlights changes made to the original version. You should upload this as a separate file labeled 'Revised Manuscript with Track Changes'.

* An unmarked version of your revised paper without tracked changes. You should upload this as a separate file labeled 'Manuscript'.

If you would like to make changes to your financial disclosure, competing interests statement, or data availability statement, please make these updates within the submission form at the time of resubmission. Guidelines for resubmitting your figure files are available below the reviewer comments at the end of this letter.

We look forward to receiving your revised manuscript.

Kind regards,

Michael Winter

Academic Editor

PLOS Digital Health

Leo Anthony Celi

Editor-in-Chief

PLOS Digital Health

orcid.org/0000-0001-6712-6626

**Journal Requirements:**

If the reviewer comments include a recommendation to cite specific previously published works, please review and evaluate these publications to determine whether they are relevant and should be cited. There is no requirement to cite these works unless the editor has indicated otherwise.

**Additional Editor Comments (if provided):**

[Note: HTML markup is below. Please do not edit.]

**Reviewers' Comments:**

Reviewer's Responses to Questions

**Comments to the Author**

1. Does this manuscript meet PLOS Digital Health’s publication criteria? Is the manuscript technically sound, and do the data support the conclusions? The manuscript must describe methodologically and ethically rigorous research with conclusions that are appropriately drawn based on the data presented.

Reviewer #1: Yes

Reviewer #2: Yes

Reviewer #3: Yes

**********

2. Has the statistical analysis been performed appropriately and rigorously?

Reviewer #1: I don't know

Reviewer #2: Yes

Reviewer #3: Yes

**********

3. Have the authors made all data underlying the findings in their manuscript fully available (please refer to the Data Availability Statement at the start of the manuscript PDF file)?

The PLOS Data policy requires authors to make all data underlying the findings described in their manuscript fully available without restriction, with rare exception. The data should be provided as part of the manuscript or its supporting information, or deposited to a public repository. For example, in addition to summary statistics, the data points behind means, medians and variance measures should be available. If there are restrictions on publicly sharing data—e.g. participant privacy or use of data from a third party—those must be specified.

Reviewer #1: Yes

Reviewer #2: Yes

Reviewer #3: Yes

**********

4. Is the manuscript presented in an intelligible fashion and written in standard English?

PLOS Digital Health does not copyedit accepted manuscripts, so the language in submitted articles must be clear, correct, and unambiguous. Any typographical or grammatical errors should be corrected at revision, so please note any specific errors here.

Reviewer #1: Yes

Reviewer #2: Yes

Reviewer #3: No

**********

5. Review Comments to the Author

Please use the space provided to explain your answers to the questions above. You may also include additional comments for the author, including concerns about dual publication, research ethics, or publication ethics. (Please upload your review as an attachment if it exceeds 20,000 characters)

Reviewer #1: This research and invention focused on overlooked area of care, and it has the potential to significantly enhance the accessibility of hearing loss diagnosis and treatment. I, therefore, understand the significance of this paper. The paper is crafted carefully and the experiments were conducted systematically and thoroughly. However, there are critical issues that are missed in this paper. First, order or presentation make the paper difficult to understand and fully evaluate. the methodology should come before the results, discussion and conclusion, which makes reading and comprehension of the paper easier. However, on this paper the methodology was the last part of the paper. Second, the sample technique wasn't explicitly mentioned, and it is not clear what measures were taken to ensure the randomness of the sample. Third, there were arbitrary thresholds without sufficient justifications. For example, in 1-button task, 1.6 second was used to categorize miss and hit, however, there is no sufficient explanation why this number was selected and what was the investigator try to achieve by timing the tasks. In addition, subsequent tasks, such as yes/no task and 3-AFC tasks, weren't timed, but adequate justifications weren't provided why these tasks weren't timed. Fourth, the discussion section of the paper was too sparse and the findings of the experiments weren't deeply explored or explained further in the context of existing literature. I also think the diversity of the participants, real clinical setting testing and the use of multi-dimensional protocols was the strength of the paper.

Reviewer #2: Thank you for giving me the opportunity to review your article.

1. The authors mentioned that there were "Some insights emerged, such as

perceived differences in test difficulty and length among candidates, and a difference in

results based on attribute wording which had a strong and clear significance in user

evaluations." (line 331). It might be worth elaborating if these differences were the result of candidates having different hearing/cognitive abilities, or if the distribution of candidates who have different experiences in test difficulty and length is similar across the different hearing/cognitive abilities groups.

2. The authors mentioned that the participants had an option to "Call the examiner: The user

always had the option to call the examiner for task explanation". It might be worth stating whether any participants called the examiner for help, and if it the calls for help affected the rating of the test experience.

Reviewer #3: This paper develops and evaluates user-operated alternatives to the ACT test. While it is relevant to PLOS Digital Health and the research overall does seem sound, I have several significant issues with the work that I don’t think can be addressed with a revision. Specifically, I think the paper needs significant rewrites, and additional experiments need to be run before I can recommend the paper for publication. Please note that the examples I use to illustrate my points are not exhaustive; there are usually more cases of the issues I point out.

Poor writing and organization:

First, this paper is rather difficult to follow at both a structural and a sentence level. Regarding the latter, while sentences are not grammatically incorrect, there is a lot of awkward phrasing that makes for a choppy and, at times, verbose read. Just taking the sentence beginning at the end of line 409 as an example, “We aimed at a total of 30 participants where there would be a control group formed of 6 young NH listeners (<30 years old), a group of people with hearing loss and with high cognitive abilities (i.e., higher scores in the RDS test, HIhi.), and another group of participants with hearing loss with lower cognitive abilities (i.e., lower scores in the RDS test HIlo)” could much more cleanly rephrased as “We aimed to recruit 30 participants for this study. This comprised 6 NH listeners, 12 listeners with hearing loss and high cognitive abilities (Hihi), and 12 listeners with hearing loss and low cognitive abilities (Hilo). Cognitive ability was determined based on RDS scores, with the high group scoring between 14 and 21 and the low group scoring between 6 and 12.”

Secondly, definitions for concepts that need to be explained frequently come after their use, so I had to read the sentence, find the definition, then go back and read the sentence again to understand what the authors were getting at. One example is lines 46-52, where the authors introduce the ACT test as a version of the STM test, neither of which is really introduced or defined (more on this later). They both involve speech reception thresholds, which are defined in the next sentence, meaning that a reader has to start this whole section over again to understand what they are reading, with the context from the later sentences. As another example, on line 91, RDS scores are introduced, but it's only in the Methods that they are noted as a test for cognitive ability (lines 407-408). As a final example here, the RAMES framework is mentioned for the first time on line 356, and even the acronym is not spelled out until line 391, where it’s just cited and not defined. I bring up these sentence-level issues to highlight the structural issues with the paper, namely that it’s not particularly accessible to readers who aren’t intimately familiar with this specific area of research. In general, it seems as though the authors struggled to write a paper where the results preceded the methods, as I frequently had to go to the Methods section to understand what was being presented in the results (this is highlighted by the fact that several times the authors stated “(see Methods section)” after portions of the Results section) and the Results section is frequently bloated by half descriptions of the Methods employed to achieve the Results. As examples, lines 87-90, 91-93, 116-122, and the entire section starting from 146 are Methods, not results. The authors need to go back and firmly delineate what goes into Methods and what goes into Results.

In general, I feel like the paper needs a significant rewrite for clarity. In particular, I would recommend that the authors read other papers that place the Results section before the Methods section and pay particular attention to how information is introduced and which sections contain which information. One particular point of emphasis: the authors really should clearly introduce the ACT and STM tests in the introduction, as these are foundational to their work, and without a clear understanding of those, readers who are not familiar with them are going to have a frustrating experience.

Study population issues:

I also have issues with the study population. I can understand why the population of hearing-impaired participants is low, as I can see how that would be a more difficult population to recruit. However, there are only 6 individuals who comprise the control group, and the only requirement for being a part of the control group is that they are below 30 years old and do not have hearing loss. There’s no reason for this group of participants to be that low, especially given that this study was conducted in a university setting. The authors should conduct the study with additional control group participants and re-run their analysis. Furthermore, is age just a proxy for hearing ability? Was one of the requirements for being in the hearing impaired group being over 50, and one of the requirements for being in the normal hearing group being under 30? Or was this just how recruitment panned out? The authors should clarify whether they recruited specifically for age or not, and if they did, they should justify why they did so.

Also, I have several issues with the inclusion of cognitive abilities. Why was this a point of emphasis? The authors should justify this with citations in the introduction. Also, why are RDS scores a fair evaluation of cognitive abilities? To me, this is a rather narrow view of cognitive ability, and there are many other tests; the authors need to justify the use of this.

A note on the language here. The statement “Since one of the study's goals was to obtain an evaluation from a diverse group of participants with varying hearing and cognitive abilities...” is confusing to me. Which of the five goals the authors laid out does this refer to? My best guess is Goal 4, but if that’s the case, evaluation on a diverse group of participants is a means of ensuring Goal 4; the goal itself is not to evaluate on a diverse group of participants. Really, I think that whole sentence can be removed, and the section can simply begin with something like “Figure 1 shows the results from…” which removes some of the Methods language from the Results section as I noted earlier. Also, the statement “We aimed at a total of 30 participants where there would be a control group formed by 6 young NH listeners” (lines 409-411) is confusing. Why did the authors aim to recruit only 30 participants? Why specifically 6 participants? Is this just a language issue, where a statement such as “We recruited a total of 30 participants that comprised 6 young NH listeners…” is what the authors meant?

Introduction issues:

The introduction generally lacks citations. There are only 10 citations throughout, and several claims really should be backed up with citations. For instance, this sentence: “The currently used audiometric tests, which are time-consuming and require active participation of a hearing care professional, represent a bottleneck that effectively limits access to hearing healthcare.” (lines 26-28), have health care professionals or patients noted that they are time consuming and represent a bottleneck? Also, this sentence: “However, there has been a growing focus on additionally evaluating supra-threshold abilities, such as the patient’s ability to discriminate speech in the presence of noise when the speech is fully audible.” (lines 38-40), are there any statistics/studies to show this shift in focus? Also, this sentence: “This has motivated the search for alternative tests capable of capturing the fundamental abilities employed in challenging 'cocktail-party’ scenarios, which are characterized by multiple interfering sound sources including speech.” (lines 43-45), are there other studies that have developed and evaluated alternative tests? Also, I think it might make the introduction stronger if the first sentence was separated into multiple sentences; the authors could include actual statistics on the scope and impact of hearing loss to really emphasize the need for improving accessibility and reducing cost.

Figure issues:

I don’t think the combination of the text, Figure 3, and Figure 3’s caption does a good job of explaining the differences between the test candidates. The authors should rework these to make it clearer what is changing between each candidate. In particular, I was looking to see if only one thing had changed between candidates, to understand what variable was being tested with each candidate, and it seems like that’s the case? I found this hard to follow.

For Figures 5-6, there’s quite a bit going on, and a lot of significance between various groups. Fundamentally, I don’t understand why positive and negative wordings were used; I appreciate that phrasing of statements can affect ratings, but there’s no meaningful follow-up on how this should influence how I interpret participants’ views on the candidates. For example, if I’m understanding correctly, in that first panel in Figure 5, there is a significant difference in the difficulty of candidates 2 and 3 when negative wording is used, but the difference is not significant when positive wording is used. So, is candidate 2 better in terms of difficulty? It just seems like these figures are introducing a lot of complexity, and all that the authors are getting out of it is a muddier picture. I will note that the point of a Discussion section is to contextualize results, and I was looking for some clarity there, but I did not find it. Also, I don’t understand what the word “wording” in the bottom left of each panel means. It also seems to cover up some data points in some panels.

Other issues:

Line 84: “A diverse group of participants” is a weird title for a subsection. I would recommend rephrasing to something like “Study participants”

Lines 87-93, the language is a bit confusing here. Initially, I read it as 28 HI participants were recruited, and an additional 6 NH participants were recruited, which made the division into 11 and 11 confusing. Please clean up the language here.

Figure 1 would be cleared if Right and Left were Right Ear and Left Ear.

Line 105, “two groups” should be “two HI groups”

Line 193, how many users were used for this refinement? These users were separate from the 28 participants that were used to evaluate the system, right?

Line 435, what iPad model was used?

**********

6. PLOS authors have the option to publish the peer review history of their article (what does this mean?). If published, this will include your full peer review and any attached files.

**Do you want your identity to be public for this peer review?**  If you choose “no”, your identity will remain anonymous but your review may still be made public.

For information about this choice, including consent withdrawal, please see our Privacy Policy.

Reviewer #1: No

Reviewer #2: No

Reviewer #3: No

**********

[NOTE: If reviewer comments were submitted as an attachment file, they will be attached to this email and accessible via the submission site. Please log into your account, locate the manuscript record, and check for the action link "View Attachments". If this link does not appear, there are no attachment files.]

**Figure resubmission:**

While revising your submission, we strongly recommend that you use PLOS’s NAAS tool (https://ngplosjournals.pagemajik.ai/artanalysis) to test your figure files. NAAS can convert your figure files to the TIFF file type and meet basic requirements (such as print size, resolution), or provide you with a report on issues that do not meet our requirements and that NAAS cannot fix.

After uploading your figures to PLOS’s NAAS tool - https://ngplosjournals.pagemajik.ai/artanalysis, NAAS will process the files provided and display the results in the "Uploaded Files" section of the page as the processing is complete. If the uploaded figures meet our requirements (or NAAS is able to fix the files to meet our requirements), the figure will be marked as "fixed" above. If NAAS is unable to fix the files, a red "failed" label will appear above. When NAAS has confirmed that the figure files meet our requirements, please download the file via the download option, and include these NAAS processed figure files when submitting your revised manuscript.

**Reproducibility:**

To enhance the reproducibility of your results, we recommend that authors of applicable studies deposit laboratory protocols in protocols.io, where a protocol can be assigned its own identifier (DOI) such that it can be cited independently in the future. Additionally, PLOS ONE offers an option to publish peer-reviewed clinical study protocols. Read more information on sharing protocols at https://plos.org/protocols?utm_medium=editorial-email&utm_source=authorletters&utm_campaign=protocols

# Author comment: Author response to Decision Letter 1

**DOI:** 10.1371/journal.pdig.0000975.r002

29 Jan 2026

Attachment Submitted filename: Response Letter_R1.pdf

# Peer review: Decision Letter 1

**DOI:** 10.1371/journal.pdig.0000975.r003

**Authors:** Zhao Ni, Michael Winter, Zhao Ni, Michael Winter

30 Mar 2026

PDIG-D-25-00510R1

-->Evaluating user-operated tests of audible contrast sensitivity towards efficient hearing healthcare services

-->PLOS Digital Health

-->Dear Dr. Sanchez-Lopez,

-->Thank you for submitting your manuscript to PLOS Digital Health. After careful consideration, we feel that it has merit but does not fully meet PLOS Digital Health's publication criteria as it currently stands. Therefore, we invite you to submit a revised version of the manuscript that addresses the points raised during the review process.

-->Please submit your revised manuscript by Apr 29 2026 11:59PM. If you will need more time than this to complete your revisions, please reply to this message or contact the journal office at digitalhealth@plos.org. When you're ready to submit your revision, log on to https://www.editorialmanager.com/pdig/ and select the 'Submissions Needing Revision' folder to locate your manuscript file.

-->Please include the following items when submitting your revised manuscript:

-->* A letter that responds to each point raised by the editor and reviewer(s). You should upload this letter as a separate file labeled 'Response to Reviewers'. This file does not need to include responses to any formatting updates and technical items listed in the 'Journal Requirements' section below.

-->* A marked-up copy of your manuscript that highlights changes made to the original version. You should upload this as a separate file labeled 'Revised Manuscript with Track Changes'.

-->* An unmarked version of your revised paper without tracked changes. You should upload this as a separate file labeled 'Manuscript'.

-->If you would like to make changes to your financial disclosure, competing interests statement, or data availability statement, please make these updates within the submission form at the time of resubmission. Guidelines for resubmitting your figure files are available below the reviewer comments at the end of this letter.

-->We look forward to receiving your revised manuscript.

-->Kind regards,

-->Michael Winter

-->Academic Editor

-->PLOS Digital Health

-->Leo Anthony Celi

-->Editor-in-Chief

-->PLOS Digital Health

-->orcid.org/0000-0001-6712-6626



**Journal Requirements:**

-->If the reviewer comments include a recommendation to cite specific previously published works, please review and evaluate these publications to determine whether they are relevant and should be cited. There is no requirement to cite these works unless the editor has indicated otherwise.

**Additional Editor Comments (if provided):**





-->[Note: HTML markup is below. Please do not edit.]

**Reviewers' Comments:**

-->Reviewer's Responses to Questions

**Comments to the Author**

1. If the authors have adequately addressed your comments raised in a previous round of review and you feel that this manuscript is now acceptable for publication, you may indicate that here to bypass the “Comments to the Author” section, enter your conflict of interest statement in the “Confidential to Editor” section, and submit your "Accept" recommendation.

Reviewer #1: All comments have been addressed

Reviewer #2: (No Response)

Reviewer #3: (No Response)

**********

2. Does this manuscript meet PLOS Digital Health’s publication criteria? Is the manuscript technically sound, and do the data support the conclusions? The manuscript must describe methodologically and ethically rigorous research with conclusions that are appropriately drawn based on the data presented.

Reviewer #1: Yes

Reviewer #2: (No Response)

Reviewer #3: Yes

**********

3. Has the statistical analysis been performed appropriately and rigorously?

Reviewer #1: I don't know

Reviewer #2: Yes

Reviewer #3: Yes

**********

4. Have the authors made all data underlying the findings in their manuscript fully available (please refer to the Data Availability Statement at the start of the manuscript PDF file)?

The PLOS Data policy requires authors to make all data underlying the findings described in their manuscript fully available without restriction, with rare exception. The data should be provided as part of the manuscript or its supporting information, or deposited to a public repository. For example, in addition to summary statistics, the data points behind means, medians and variance measures should be available. If there are restrictions on publicly sharing data—e.g. participant privacy or use of data from a third party—those must be specified.

Reviewer #1: Yes

Reviewer #2: Yes

Reviewer #3: Yes

**********

5. Is the manuscript presented in an intelligible fashion and written in standard English?

PLOS Digital Health does not copyedit accepted manuscripts, so the language in submitted articles must be clear, correct, and unambiguous. Any typographical or grammatical errors should be corrected at revision, so please note any specific errors here.

Reviewer #1: Yes

Reviewer #2: Yes

Reviewer #3: (No Response)

**********

6. Review Comments to the Author

Please use the space provided to explain your answers to the questions above. You may also include additional comments for the author, including concerns about dual publication, research ethics, or publication ethics. (Please upload your review as an attachment if it exceeds 20,000 characters)

Reviewer #1: The structure of the paper is improved significantly, enhancing readability of the study. Sentence level issues are also resolved, and enabled easy understanding of the paper. Compelling clarifications were provided with ample citations which strengthened the discussions on the paper. The technical parts are also adequately explained.

Reviewer #2: I would like to thank the authors for their thoughtful and constructive response to the previous round of peer review.

An aim of the study is to ensure that "the test should be accessible to a broad group of patients without requiring any special modifications for subpopulations, including those with low cognitive abilities.", Upon reviewing the section on participants recruited, there are participants who are hearing-impaired with both higher and lower cognitive abilities. However there seems to be only participants with normal hearing and higher cognitive abilities, and there seems to be a lack of participants with normal hearing abilities and lower cognitive abilities. It would be good if the authors could elaborate on the choice of participants, especially as a key strength of the study mentioned was inclusion of "a diverse participant selection regarding hearing losses and cognitive abilities".

Thank you!

Reviewer #3: The authors have done an excellent job of responding to reviewer feedback and making appropriate revisions. I'm generally satisfied with the updated manuscript, but I do have a couple of comments.

1. While I appreciate the thought behind adding a new section after the introduction and before the results, it is effectively a methods section, and it is a bit strange to have two methods sections. I will note that while PLOS's submission guidelines do state that the preference is for the Methods section to come after the Discussion, it does state "To provide flexibility, however, authors are also able to include the Materials and Methods section before the Results section or before the Discussion section." Given there's quite a bit of context required to follow the Results section, I might recommend just combining the "Experimental design and study framework" section and "Methods" section into one section that follows the Introduction and precedes the Results. The Methods section is also quite long, and I would recommend considering moving non-essential definitions to supplemental materials. If the authors really would like to keep the methods after the discussion, I don't think adding a pseudo-methods section is the way to go; rather, the authors should work in the needed context into the introduction/results section.

2. A suggestion to improve readability: At the end of the introduction, the authors lay out five objectives, but these aren't referred to again after that. Given the number of results, it may help to refer back to these objectives in the results (and maybe also the discussion) so readers can quickly understand what objectives are being addressed by each finding. With this approach, I'd also recommend making the list of objectives in the introduction into an actual numbered list to give it the visual standout so that readers know to pay attention to it.

**********

7. PLOS authors have the option to publish the peer review history of their article (what does this mean?). If published, this will include your full peer review and any attached files.

**Do you want your identity to be public for this peer review?**  If you choose “no”, your identity will remain anonymous but your review may still be made public.

For information about this choice, including consent withdrawal, please see our Privacy Policy.

Reviewer #1: No

Reviewer #2: No

Reviewer #3: No

**********

-->[NOTE: If reviewer comments were submitted as an attachment file, they will be attached to this email and accessible via the submission site. Please log into your account, locate the manuscript record, and check for the action link "View Attachments". If this link does not appear, there are no attachment files.]

**Figure resubmission:**

-->While revising your submission, we strongly recommend that you use PLOS’s NAAS tool (https://ngplosjournals.pagemajik.ai/artanalysis) to test your figure files. NAAS can convert your figure files to the TIFF file type and meet basic requirements (such as print size, resolution), or provide you with a report on issues that do not meet our requirements and that NAAS cannot fix.

After uploading your figures to PLOS’s NAAS tool - https://ngplosjournals.pagemajik.ai/artanalysis, NAAS will process the files provided and display the results in the "Uploaded Files" section of the page as the processing is complete. If the uploaded figures meet our requirements (or NAAS is able to fix the files to meet our requirements), the figure will be marked as "fixed" above. If NAAS is unable to fix the files, a red "failed" label will appear above. When NAAS has confirmed that the figure files meet our requirements, please download the file via the download option, and include these NAAS processed figure files when submitting your revised manuscript.

**Reproducibility:**

-->To enhance the reproducibility of your results, we recommend that authors of applicable studies deposit laboratory protocols in protocols.io, where a protocol can be assigned its own identifier (DOI) such that it can be cited independently in the future. Additionally, PLOS ONE offers an option to publish peer-reviewed clinical study protocols. Read more information on sharing protocols at https://plos.org/protocols?utm_medium=editorial-email&utm_source=authorletters&utm_campaign=protocols

# Author comment: Author response to Decision Letter 2

**DOI:** 10.1371/journal.pdig.0000975.r004

23 Apr 2026

Attachment Submitted filename: Response Letter_R2.pdf

# Editor report: Decision Letter 2

**DOI:** 10.1371/journal.pdig.0000975.r005

**Authors:** Zhao Ni, Michael Winter, Zhao Ni, Michael Winter, Zhao Ni, Michael Winter

16 Jun 2026

Multi-dimensional evaluation of user-operated audible contrast sensitivity tests towards efficient hearing healthcare services

PDIG-D-25-00510R2

Dear Sanchez-Lopez,

We are pleased to inform you that your manuscript 'Multi-dimensional evaluation of user-operated audible contrast sensitivity tests towards efficient hearing healthcare services ' has been provisionally accepted for publication in PLOS Digital Health.

Before your manuscript can be formally accepted you will need to complete some formatting changes, which you will receive in a follow-up email from a member of our team.

Please note that your manuscript will not be scheduled for publication until you have made the required changes, so a swift response is appreciated.

IMPORTANT: The editorial review process is now complete. PLOS will only permit corrections to spelling, formatting or significant scientific errors from this point onwards. Requests for major changes, or any which affect the scientific understanding of your work, will cause delays to the publication date of your manuscript.

If your institution or institutions have a press office, please notify them about your upcoming paper to help maximize its impact. If they'll be preparing press materials, please inform our press team as soon as possible -- no later than 48 hours after receiving the formal acceptance. Your manuscript will remain under strict press embargo until 2 pm Eastern Time on the date of publication. For more information, please contact digitalhealth@plos.org.

Thank you again for supporting Open Access publishing; we are looking forward to publishing your work in PLOS Digital Health.

Best regards,

Michael Winter

Academic Editor

PLOS Digital Health

***********************************************************

**Additional Editor Comments (if provided):**

**Reviewer Comments (if any, and for reference):**
